# The Registry of Senior Australians: Informing Aged Care Policy Reforms.

**DOI:** 10.23889/ijpds.v7i3.1862

**Published:** 2022-08-25

**Authors:** Gillian Caughey, Olivia Ryan, Maria Crotty, Craig Whitehead, Susan Hillier, Keith Evans, Marilyn Von Thien, Cassie Mason, Chris Radbone, Jane Mussared, Julie Ratcliffe, Sally Tideman, Anna Barker, Anna Sheppeard, Caroline Miller, Odette Pearson, Megan Corlis, Allen Candy, Steve Wesselingh, Maria Inacio

**Affiliations:** 1 Registry of Senior Australians, South Australian Health and Medical Research Institute; 2 Flinders University; 3 University of South Australia; 4 Consumer Representative; 5 South Australian Department of Health; 6 SA/NT Data Link; 7 Council of the Ageing, SA; 8 ECH Inc; 9 Silver Chain Aged Care; 10 South Australian Health and Medical Research Institute; 11 Life Care

## Objectives

To: (1) outline the research produced using linked data from the Registry of Senior Australians (ROSA) which informed the recommendations from Australia’s Royal Commission into Aged Care Quality and Safety (delivered February 2021); (2) describe the Australian Government Aged Care Roadmap Reforms (announced May 2021) resulting from the recommendations.

## Approach

ROSA was established in 2017 and is led by a partnership of scientists, clinicians, aged care providers and consumer advocates from nine organisations seeking to improve the lives of Australians in aged care. ROSA is a Clinical Quality Registry comprised of linked national and cross-jurisdictional aged and health care data and includes a national historical de-identified cohort (3.5 million individuals, 2002-2020) and a prospectively enrolled cohort in the state of South Australia (26,600 individuals, 2018-current). This is a summary of ROSA’s high-quality evidence used by the Royal Commission and translation of this evidence into policy by leveraging existing data infrastructure.

## Results

Between 2019-2020 the ROSA team led the delivery of four in-depth reports for the Royal Commission, contributed data and expertise to an additional four published Commission reports. Examples of ROSA outputs informing the Commissions’ recommendations included: evidence of national increased psychotropic medication use following entry to residential aged care, evidence of higher risk of mortality and entry to permanent care while waiting for home care packages, development of quality indicators to monitor quality and safety of care nationally, and to facilitate international comparisons and benchmarking. Examples of recommendations included in the Australian Government Aged Care Roadmap: release of substantial funding to increase the availability of home care packages, public reporting system for quality and safety monitoring and several changes to medication management.

## Conclusion and Relevance

Registries are key resources for high quality real-world evidence generation needed to inform national investigations, ultimately leading to significant sector reform. The ROSA experience highlights that cross-sectoral data linkages, together with technical expertise, informed by clinicians and consumers, are invaluable resources for system reform and policy generation.

